# Clustering and Beamwidth Optimization for UAV-Assisted Wireless Communication

**DOI:** 10.3390/s23239614

**Published:** 2023-12-04

**Authors:** Weidong Zhao, Jun Zhang, Dongxing Li

**Affiliations:** 1 College of Telecommunication and Information Engineering, Nanjing University of Posts and Telecommunications, Nanjing 210003, China; zhaoweidong@chzc.edu.cn (W.Z.); 1220013326@njupt.edu.cn (D.L.); 2 Information Engineering College, Chuzhou Polytechnic, Chuzhou 239000, China

**Keywords:** UAV wireless communications, ground user clustering, beamwidth, energy efficiency

## Abstract

With the development of wireless communication technology, unmanned aerial vehicles (UAV) are now widely used in many complex communication scenarios. When a UAV serves as an aerial base station for urban and rural ground users or marine users, it is necessary to consider the clustering of ground users and the energy efficiency of the UAV since the users are usually randomly distributed. For the scenario with randomly distributed ground users and different densities of ground users in urban and rural areas, a clustering and beamwidth optimization method for UAV-assisted wireless communication is proposed. Firstly, the energy efficiency expression of a UAV serving ground users was derived in a downlink wireless communication system assisted by a UAV. Secondly, based on the geographical location information of non-uniformly distributed users, an improved k-means method is proposed to cluster ground users, ensuring that the number of users in each cluster is within an appropriate range. Then, based on the clustering results, a fixed-point iteration (FPI) algorithm was proposed to design the optimal beamwidth of UAVs and improve their energy efficiency. Finally, the superiority of the proposed algorithm in improving energy efficiency was verified through simulation analysis, and the impact of parameters such as the cluster number and transmission power on system energy efficiency was also analyzed.

## 1. Introduction

Unmanned aerial vehicles (UAV) are unmanned vehicles controlled by a radio remote control unit and local programs. UAVs have been applied to various technological fields, including wireless communication, sensing, information processing, intelligent controls, and aviation propulsion. The key value of UAVs’ use lies in the expanded airborne platform combined with other components, thus replacing human operation in the air [1].

The technologies pertinent to UAVs have experienced rapid progress in recent years. Benefitting from their high mobility and stable operability, UAV can provide fundamental communication services even in remote areas or under severe natural disasters. As a result, UAV-assisted wireless communication has been widely applied in the academic and industrial fields [2,3,4]. On the one hand, UAVs can be used as a wireless relay to enhance the connectivity of ground wireless equipment and expand the network coverage. On the other hand, they can also be used as a mobile aerial base station to provide reliable downlink and uplink communication for the ground users to increase the capacity of wireless networks [5,6,7]. Furthermore, UAVs’ mobility and the self-adaptive communication scheme can be jointly designed to further improve the communication performance. Therefore, UAV-assisted communication is an essential part of the future wireless communication system, with promising developments [8].

Among various studies on UAV-assisted wireless communication, most of the results have been obtained based the hypothesis of a uniform user distribution on the ground and the same flight altitude of the UAV. For static ground users, the authors put forward the optimal UAV deployment in [9] based on the premise of the satisfaction of the user rate requirement. In [10], the flight altitude of the UAV is taken into account to increase the throughput of the communication system by jointly optimizing the UAV’s flight altitude and beamwidth. A joint optimization algorithm for user grouping and power allocation in an UAV-aided non-orthogonal multiple access (NOMA) system is proposed in [11]. The authors construct the problem of resource allocation for a multi-user NOMA system to maximize the sum-rate, where the maximum transmitted power constraint at the base station and the user grouping constraint are both considered. In [12], the authors study the control of uplink power. With the minimum rate requirement, the total power is minimized by jointly taking the altitude, beamwidth, geographic location, and bandwidth allocation into account.

The assumption of the uniform user distribution is commonly adopted; however, in reality, the user distribution may not be uniform for most conditions. For instance, the user density between urban and rural areas varies and the user distribution in the marine environment is also related to the route. In addition, the energy consumption of UAV is also a focus that cannot be neglected. As a matter of fact, UAV has limited energy for transmission, maneuvering, control, data processing, and payload purposes. Its energy consumption is also impacted by the UAV’s role, flight mission, weather condition, and navigation path. The UAV energy limitation is of significant importance for UAVs’ deployment and maneuvering in various applications. Thus, the energy efficiency of a UAV should be carefully considered, since the energy-saving strategy in the remote communication has a great impact on the communication system [13].

In this paper, a user clustering and beamwidth optimization method for UAV-assisted wireless communication is proposed. Based on setting the restrictions on the number of users in each cluster, the ground users are divided into several clusters according to their geographic locations, and the UAVs are used to transmit data to the ground users in the cluster.

The contributions of this paper are as follows:

1. A UAV-assisted downlink wireless communication system is established for the scenario of a UAV serving non-uniformly distributed ground users as an air base station, and the energy efficiency expression of a UAV serving ground users is derived;
2. Based on the geographic location information of non-uniformly distributed users, an improved k-means method is proposed to cluster ground users, ensuring that the number of users in each cluster is within an appropriate range. Then, based on the clustering results, a fixed-point iteration (FPI) algorithm is proposed to design the optimal beamwidth of UAVs. This method can effectively improve the energy efficiency of UAVs serving ground users;
3. Through simulation analysis, the differences in UAV energy efficiency between the fixed beamwidth and random clustering scenarios in other studies are compared, verifying the superiority of the proposed algorithm in improving energy efficiency. At the same time, the influence of parameters such as the cluster number and transmission power on system energy efficiency is also analyzed.

## 2. System Model and Problem Formulation

### 2.1. System Model

**Figure 1:**
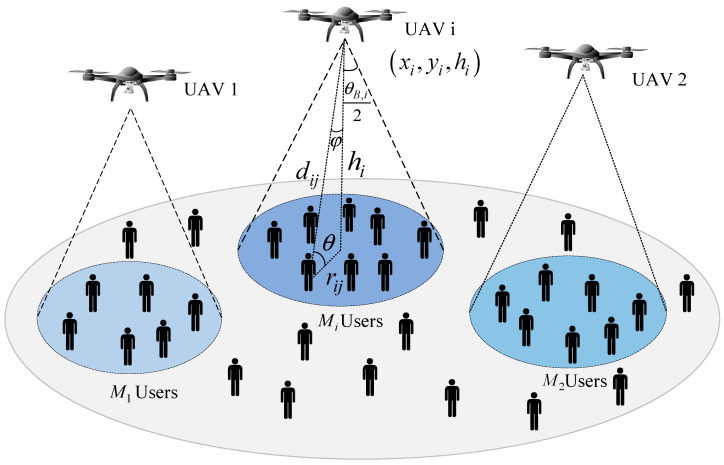
UAV-assisted wireless communication system model.

We consider the UAV-assisted downlink wireless communication system shown in Figure 1. The mentioned system consists of N UAVs and M users on the ground. These UAVs are equipped with directional antennas with an adjustable beamwidth, and each user on the ground has an omnidirectional antenna with unit gain. Furthermore, the M users on the ground are non-uniformly distributed. The UAVs can serve as a mobile base station in the air and the N UAVs can conduct data transmission with the M users on the ground. Assuming that each user on the ground is within the coverage of UAV, where the number of users covered by the *i*-th UAV is $M_{i}$. To simplify the theoretical analysis and research, we assume that a UAV in the downlink scenario adopts the frequency division multiple access (FDMA) technology to carry out data transmission with the users on the ground in this paper. FDMA will distribute the specific frequency bandwidth to the users on the ground. In this way, each user can have their own dedicated channel for wireless communication. Under such conditions, the frequency interference between adjacent UAVs can be negligible. In Figure 1, a target UAV and its target users within its coverage radius are first selected for analysis.

A 3D coordinate is set up by taking the projection in the area for the target UAV. We assume that the locations of UAV i and user j on the ground are expressed as $\left(x_{i},y_{i},h_{i}\right)$ and $\left(x_{j},y_{j},0\right)$, respectively, where $h_{i}$ stands for the flight altitude of UAV i and $r_{ij}=\sqrt{{\left(x_{i}-x_{j}\right)}^{2}+{\left(y_{i}-y_{j}\right)}^{2}}$ signifies the horizontal distance from the UAV to the users on the ground. In the section, $\theta_{B,i}$ is utilized to represent the directional antenna with half power beamwidth equipped on UAV i, and G signifies the antenna gain of a directional antenna [14], which can be specifically expressed as

(1) $$G=\left\{\begin{array}{l} G_{3dB}\;,\;-\frac{\theta_{B}}{2}\;\leq \;\varphi \;\leq \;\frac{\theta_{B}}{2}\;\\ g\left(\varphi \right)\;,\;\mathrm{else}. \end{array}\right.$$

where φ refers to the angle of the sector, $G_{3dB}\approx 30000/\theta_{B}^{2}$ stands for the main lobe gain ($\theta_{B}$ is in degree), and $g\left(\varphi \right)$ signifies the antenna gain existed outside the main lobe with an extremely small value, which is neglectable.

The extensively applied air-to-ground path loss model proposed in [6] and [15] is adopted in this paper. According to existing research, the communication user group is composed of two parts, which are the receiver with a line-of-sight (LoS) connection and the others with a non-line-of-sight (NLoS) connection. The latter can still receive the signals from the receiver due to the strong reflection and diffraction. The essential factor for the channel model is the possibility to establish an LoS connection between the transmitter and the receiver. Furthermore, the probability of LoS (denoted by $P\left(\mathrm{LoS}\right)$) depends on the considered environment (such as a rural area, an urban area or others) and the direction of the UAV and users on the ground. In [6,15], the probability of LoS connection is expressed as

(2) $$P\left(\mathrm{LoS}\right)=\frac{1}{1+\alpha \exp\left[-\beta \left(\theta -\alpha \right)\right]},$$

where α and β refer to the parameters with constant values depending on the particular environment, and $\theta =\arctan(h_{i}/r_{ij})$ stands for the elevation angle from users to UAV.

According to the free space propagation model, there are two links for the data transmission between a UAV and users on the ground, i.e., LoS and NLoS. In addition, each link has its specific probability of occurrence. The path loss of the LoS and NLoS link in the air-to-ground communication can, respectively, be expressed as [16]

(3) $$PL_{\mathrm{LoS}/\mathrm{NLoS}}=\left\{\begin{array}{l} {\left(\frac{4\pi f_{c}d_{ij}}{c}\right)}^{\gamma }\eta_{\mathrm{LoS}},\;\mathrm{LoS}\;\mathrm{link};\;\\ {\left(\frac{4\pi f_{c}d_{ij}}{c}\right)}^{\gamma }\eta_{\mathrm{NLoS}},\;\mathrm{NLoS}\;\mathrm{link}\;,\; \end{array}\right.$$

where $f_{c}$ is the carrier frequency, c is the velocity of light, γ refers to the path loss index, $d_{ij}$ signifies the distance between the UAV and the users, and $\eta_{\mathrm{LoS}}$ and $\eta_{\mathrm{NLoS}}$, respectively, refer to the extra path loss that increased due to the free space propagation from the LoS and NLoS link. In this paper, γ is taken as 2 for simplicity. Obviously, in terms of the NLoS link, its loss is higher than that of the LoS link due to the existence of shadow and diffraction losses in the propagation path. In conclusion, the average path loss [17] of the entire air-to-ground wireless communication can be expressed as

(4) $$\begin{array}{cc} PL & =PL_{\mathrm{LoS}}P(\mathrm{LoS})+PL_{\mathrm{NLoS}}P(\mathrm{NLoS})\\ & =PL_{\mathrm{LoS}}P(\mathrm{LoS})+PL_{\mathrm{NLoS}}\left[1-P(\mathrm{LoS})\right]\\ & ={\left(\frac{4\pi f_{c}d_{ij}}{c}\right)}^{2}\left[\eta_{\mathrm{NLoS}}+P(\mathrm{LoS})\left(\eta_{\mathrm{LoS}}-\eta_{\mathrm{NLoS}}\right)\right]. \end{array}$$

Considering the communication between the UAV i and the users j on the ground in Figure 1, the power transmitted from UAV i to the users on the ground within its coverage range is $P_{t}$, which is allocated equally to the users within the coverage. The corresponding reachable rate expression can be obtained as

(5) $$\begin{array}{cc} R_{ij} & =\frac{B}{M_{i}}\log_{2}\left(1+\frac{\frac{P_{t}}{M_{i}}G}{PL\cdot N_{0}\frac{B}{M_{i}}}\right)\\ & =\frac{B}{M_{i}}\log_{2}\left(1+\frac{P_{t}G}{PL\cdot N_{0}B}\right), \end{array}$$

where B is the total bandwidth of UAV i, and the bandwidth is equally allocated to all users within the coverage of the UAV. As a result, the bandwidth obtained by each user is $B/M_{i}$, $N_{0}$ stands for the power spectral density, PL is the average path loss between UAV i and the user j, and G refers to the antenna gain of the directional antenna.

Consequently, in the given geographic area, the overall rate can be expressed as

(6) $$R=\sum_{i=1}^{N}\sum_{j=1}^{M_{i}}\frac{B}{M_{i}}\log_{2}\left(1+\frac{P_{t}G}{PL\cdot N_{0}B}\right),$$

where M is the total number of users in a given area, N is the total number of UAVs, and we have $\sum_{i=1}^{N}M_{i}=M$.

Then, the total power consumed by UAV in a given area is

(7) $$P_{\mathrm{total}}=N\left(P_{t}+P_{c}\right),$$

where $P_{c}$ is the onboard circuit power consumed by the UAV [18,19]. According to (6) and (7), the energy efficiency of the entire wireless communication system can be expressed as

(8) $$EE=\frac{\sum_{i=1}^{N}\sum_{j=1}^{M_{i}}A_{ij}\frac{B}{M_{i}}\log\left(1+\frac{P_{t}G}{PL\cdot N_{0}B}\right)}{N(P_{t}+P_{c})}.$$

### 2.2. Problem Formulation

The objective of this paper is to maximize the system’s energy efficiency under the given constraints to discover the optimal beam width of the directional antenna, which is given by

(9) $$\begin{array}{l} \underset{\theta_{B,i},A_{ij}}{\max}\;\;\;EE,\\ \;\;\;s.t.\;\;\;\;C1:\;\;\;\sum_{i=1}^{N}M_{i}=M,\\ \;\;\;\;\;\;\;\;\;\;\;\;\;\;C2:\;\;\;\;A_{ij}=\{0,1\},\\ \;\;\;\;\;\;\;\;\;\;\;\;\;\;C3:\;\;\;\;\sum_{i=1}^{N}A_{ij}=1,\\ \;\;\;\;\;\;\;\;\;\;\;\;\;\;C4:\;\;\;\;\sum_{j=1}^{M}A_{ij}=M_{i}\;,\;\\ \;\;\;\;\;\;\;\;\;\;\;\;\;\;C5:\;\;\;\;M_{\min}\leq M_{i}\;\leq \;M_{\max},\; \end{array}$$

where the number of total users in the given area is M, and $A_{ij}$ signifies the correlation factor. $A_{ij}$ has only two possible values: 0 and 1. In the event that $A_{ij}=1$, it means that UAV i links to the user j on the ground for data transmission. However, in the event that $A_{ij}=0$, it indicates that no UAV links to the users on the ground. C3 means that only one UAV can link to user j on the ground; C4 signifies that UAV i can only link to $M_{i}$ users on the ground within its cluster, and C5 stands for the restricted condition of the number of users on the ground $M_{i}$ within the cluster, where $M_{\min}$ represents the minimum value of the number of users in the cluster and $M_{\max}$ stands for the corresponding maximum value.

The problem in (9) is difficult to solve directly due to the non-convexity, so we divide it into two subproblems. One is the linking problem of UAV and users on the ground (namely, the user clustering problem); the other is the problem of maximizing the energy efficiency of the wireless communication system by reasonably designing the beamwidth of the directional antenna. Therefore, the next section will analyze the user clustering problem.

## 3. Ground User Clustering Method

As the constraints C2, C3, and C4 in (9) all concern the link between UAV and users on the ground. Furthermore, the users in this paper are distributed uniformly in the geographic area. Therefore, the user cluster processing should be carried out first, thus allowing the shortest distance from the UAV to the users on the ground in the cluster. The specific expression of the first subproblem is formulated as

(10) $$\begin{array}{l} \underset{A_{ij}}{\min}\sum_{j=1}^{M}\sum_{i=1}^{N}A_{ij}\left[h_{i}^{2}+{\left(x_{i}-x_{j}\right)}^{2}+{\left(y_{i}-y_{j}\right)}^{2}\right],\\ \;s.t.\;\;\;\;\;\;\;\;\;\;\;\;A_{ij}=\{0,1\}\;,\;\\ \;\;\;\;\;\;\;\;\;\;\;\;\;\;\;\;\;\;\;\sum_{i=1}^{N}A_{ij}=1\;,\;\\ \;\;\;\;\;\;\;\;\;\;\;\;\;\;\;\;\;\;\;\sum_{j=1}^{M}A_{ij}=M_{i},\\ \;\;\;\;\;\;\;\;\;\;\;\;\;\;\;\;\;\;\;M_{\min}\leq M_{i}\;\leq \;M_{\max}. \end{array}$$

The number of users on the ground in each cluster in (10) has certain restrictions. Through analysis, it can be discovered that the square of the distance from UAV to all users in the cluster is the shortest if the UAV is right above the cluster center. Thus, it is essential to conduct the clustering properly.

The k-means clustering algorithm is one of the most classical algorithms in the field of unsupervised learning. The distance is taken as the evaluation index of similarity, i.e., the closer the distance between two objects, the higher the similarity. In terms of the given sample set, the k-means algorithm can divide it into K clusters according to the adjacent distance of samples. The major requirement of clustering is to make sure that the sample points in the cluster are linked as closely as possible so that the distance can be as small as possible [20]. The rationale and the implementation of the k-means algorithm are relatively simple. It has been extensively applied due to its better clustering effect and it explainable results.

Based on the traditional k-means clustering method, this paper further improves the clustering algorithm. A maximum limit is set on the number of users in each cluster so as to ensure the relatively equally distributed number of users served by the UAV. In this way, poor communication quality can be avoided due to the excessive number of users of some of the UAVs. The specific clustering steps are shown as below:

1. Firstly, N points are randomly selected as the original centroid;
2. The distances from all users on the ground to the N centroid are calculated;
3. Clustering should be based on the distance. If a ground user is closer to a certain centroid, the user should be assigned to the cluster containing the centroid; if the distance from the user to multiple centroids are equal and the number of users on the ground does not exceed the given limit, it can be distributed to any one of these clusters. However, if the number of users on the ground in the cluster exceeds the limit, re-clustering should be carried out;
4. The mean value of users’ locations in each cluster obtained from calculation is taken as the new centroid after clustering all users;
5. Steps 2, 3 and 4 are constantly repeated and clustering is topped when the calculated new centroid is equal to the original one.

The traditional k-means clustering method may have unevenly distributed users in each cluster with a quite different number of users. Despite the different number of users on the ground obtained, the improved k-means clustering method in this paper can relatively equally distribute the users in all clusters due to the certain limit of the minimum number of users.

## 4. Energy Efficiency Analysis

According to the improved k-means clustering method in the above, the users on the ground can be divided into N clusters, and accordingly, the corresponding locations can be obtained. Then, the antenna gain of the directional antenna obtained from (1) and the average path loss from (4) are substituted into the energy efficiency in (8), and we obtain

(11) $$\begin{array}{cc} EE & =\frac{1}{N(P_{t}+P_{c})}\sum_{i=1}^{N}\sum_{j=1}^{M_{i}}\frac{B}{M_{i}}\\ & \;\cdot \log\{1+\;{\left[\eta_{\mathrm{NLoS}}+\frac{\eta_{\mathrm{LoS}}-\eta_{\mathrm{NLoS}}}{1+\alpha \exp\left[-\beta \left(\arctan\left(\frac{h_{i}}{r_{ij}}\right)-\alpha \right)\right]}\right]}^{-1}\;\times \frac{2.2846P_{t}}{{\left(\frac{\theta_{B,i}}{2}\right)}^{2}{\left(\frac{4\pi f_{c}}{c}\right)}^{2}\left(r_{ij}^{2}+h_{i}^{2}\right)N_{0}B}\}. \end{array}$$

In this paper, the maximum distance from all users on the ground to the cluster center is taken as the coverage radius of UAV. $S_{\max}\left(i\right)$ is adopted to signify the maximum distance from the users on the ground in the *i*-th cluster to the cluster center, i.e., the maximum distance for the ground user in the coverage area of the *i*-th UAV and the projection of the UAV on the ground.

According to the equation $\tan\frac{\theta_{B,i}}{2}=\frac{S_{\max}\left(i\right)}{h_{i}}$, the specific expression of the UAV’s flight altitude can be obtained, which is substituted into (11). It can be discovered from (11) that the specific location of ground users, the number of ground users in each cluster, and the various distances from UAV to ground users can be obtained when grouping the ground users with the improved k-means clustering method. With the given residual variables, the energy efficiency in (11) is only related to the beamwidth $\frac{\theta_{B,i}}{2}$. Formula (11) represents the total energy efficiency of N UAVs. To maximize the total energy efficiency, it is necessary to maximize the energy efficiency of each individual UAV. Simplifying (11) yields the energy efficiency of an individual UAV. Let $F(\frac{\theta_{B,i}}{2})$ denote the energy efficiency of UAV *i*. For the sake of convenient analysis, t is used to replace $\frac{\theta_{B,i}}{2}$ and the function $F\left(t\right)$ can be obtained as

(12) $$\begin{array}{ll} F\left(t\right) & =\frac{2.2846P_{t}c^{2}}{4\pi^{2}f_{c}^{2}\theta_{B}^{2}N_{0}B\left(r_{ij}^{2}+\frac{S_{\max}^{2}\left(i\right)}{\tan^{2}\left(\frac{\theta_{B,i}}{2}\right)}\right)}\;\;\;\;\times \left[\overset{\;}{\underset{\overset{\;}{\underset{\;}{\;}}}{\;}}\right.\eta_{\mathrm{NLoS}}+\frac{\eta_{\mathrm{LoS}}-\eta_{\mathrm{NLoS}}}{1+\alpha \exp\left(\alpha \beta \right)\exp\left(-\beta \arctan\frac{S_{\max}\left(i\right)}{\tan\left(\frac{\theta_{B,i}}{2}\right)r_{ij}}\right)}\;\;\;\;{\left.\overset{\;}{\underset{\overset{\;}{\underset{\;}{\;}}}{\;}}\right]}^{-1}\\ & \overset{t=\frac{\theta_{B,i}}{2}}{=}\frac{2.2846P_{t}c^{2}}{16\pi^{2}f_{c}^{2}t^{2}N_{0}B\left(r_{ij}^{2}+\frac{S_{\max}^{2}\left(i\right)}{\tan^{2}\left(t\right)}\right)}\;\;\times \left[\overset{\;}{\underset{\overset{\;}{\underset{\;}{\;}}}{\;}}\right.\eta_{\mathrm{NLoS}}\;+\;\;\;\frac{\eta_{\mathrm{LoS}}-\eta_{\mathrm{NLoS}}}{1+\alpha \exp\left(\alpha \beta \right)\exp\left(-\beta \arctan\frac{S_{\max}\left(i\right)}{\tan\left(t\right)r_{ij}}\right)}\;{\left.\overset{\;}{\underset{\overset{\;}{\underset{\;}{\;}}}{\;}}\right]}^{-1}. \end{array}$$

It can be discovered from (12) that $F\left(t\right)$ is only related to t, which is beamwidth of the directional antenna. The optimal beamwidth can be solved by differentiating (12), which is expressed as

(13) $$\begin{array}{cc} \frac{\partial F\left(t\right)}{\partial t} & =\frac{-2.2846P_{t}c^{2}}{16\pi^{2}f_{c}^{2}N_{0}Bt^{4}{\left(r_{ij}^{2}+\frac{S_{\max}^{2}\left(i\right)}{\tan^{2}\left(t\right)}\right)}^{2}\left[\overset{\;}{\underset{\overset{\;}{\underset{\;}{\;}}}{\;}}\right.\eta_{\mathrm{NLoS}}\;+\;\;\;\frac{\eta_{\mathrm{LoS}}-\eta_{\mathrm{NLoS}}}{1+\alpha \exp\left(\alpha \beta \right)\exp\left(-\beta \arctan\frac{S_{\max}\left(i\right)}{\tan\left(t\right)r_{ij}}\right)}\;{\left.\overset{\;}{\underset{\overset{\;}{\underset{\;}{\;}}}{\;}}\right]}^{2}}\\ & \times \left\{\left[2t\left(r_{ij}^{2}+\frac{S_{\max}^{2}\left(i\right)}{\tan^{2}\left(t\right)}\right)+t^{2}\left(S_{\max}^{2}\left(i\right)\frac{-2\cos\left(t\right)}{\sin^{3}\left(t\right)}\right)\right]\times \left[\overset{\;}{\underset{\overset{\;}{\underset{\;}{\;}}}{\;}}\right.\eta_{\mathrm{NLoS}}\;+\;\;\;\frac{\eta_{\mathrm{LoS}}-\eta_{\mathrm{NLoS}}}{1+\alpha \exp\left(\alpha \beta \right)\exp\left(-\beta \arctan\frac{S_{\max}\left(i\right)}{\tan\left(t\right)r_{ij}}\right)}\;\left.\overset{\;}{\underset{\overset{\;}{\underset{\;}{\;}}}{\;}}\right]\right.\\ & -\left.t^{2}\left(r_{ij}^{2}+\frac{S_{\max}^{2}\left(i\right)}{\tan^{2}\left(t\right)}\right)\;\frac{\left(\eta_{\mathrm{LoS}}-\eta_{\mathrm{NLoS}}\right)\alpha \exp\left(\alpha \beta \right)\exp\left(-\beta \arctan\frac{S_{\max}\left(i\right)}{\tan\left(t\right)r_{ij}}\right)}{{\left[1+\alpha \exp\left(\alpha \beta \right)\exp\left(-\beta \arctan\frac{S_{\max}\left(i\right)}{\tan\left(t\right)r_{ij}}\right)\right]}^{2}}\times \frac{\beta r_{ij}S_{\max}\left(i\right)}{S_{\max}^{2}\left(i\right)\cos^{2}\left(t\right)+\sin^{2}\left(t\right)r_{ij}^{2}}\right\}. \end{array}$$

When $\frac{\partial F\left(t\right)}{\partial t}=0$, the optimal beamwidth maximizing the energy efficiency of the entire wireless communication system can be determined. However, it is difficult to obtain a closed-form solution of t by analyzing the above equation. However, $\frac{\partial F\left(t\right)}{\partial t}=0$ can be converted into $t=g\left(t\right)$, and by setting an initial value and iterating through $t=g\left(t\right)$, we can obtain the value of t when $\frac{\partial F\left(t\right)}{\partial t}=0$. By rearranging (13) for $\frac{\partial F\left(t\right)}{\partial t}=0$, the specific expression of $g\left(t\right)$ can be obtained as follows.

(14) $$\begin{array}{cc} g\left(t\right) & =\frac{t^{2}S_{\max}^{2}\left(i\right)\cos\left(t\right)}{r_{ij}^{2}\sin^{3}\left(t\right)+S_{\max}^{2}\left(i\right)\sin\left(t\right)\cos^{2}\left(t\right)}+\frac{\alpha \exp\left(\alpha \beta \right)\exp\left(-\beta \arctan\frac{S_{\max}\left(i\right)}{\tan\left(t\right)r_{ij}}\right)}{2\left[1+\alpha \exp\left(\alpha \beta \right)\exp\left(-\beta \arctan\frac{S_{\max}\left(i\right)}{\tan\left(t\right)r_{ij}}\right)\right]}\\ & \times \frac{t^{2}\left(\eta_{\mathrm{LoS}}-\eta_{\mathrm{NLoS}}\right)}{\left\{\eta_{\mathrm{NLoS}}\;\left[1+\alpha \exp\left(\alpha \beta \right)\exp\left(-\beta \arctan\frac{S_{\max}\left(i\right)}{\tan\left(t\right)r_{ij}}\right)\right]+\;\;\;\left(\eta_{\mathrm{LoS}}-\eta_{\mathrm{NLoS}}\right)\right\}}\times \frac{\beta r_{ij}S_{\max}\left(i\right)}{S_{\max}^{2}\left(i\right)\cos^{2}\left(t\right)+\sin^{2}\left(t\right)r_{ij}^{2}}. \end{array}$$

Based on the principle of “energy efficiency maximization”, the optimal $t^{opt}$ can be obtained through Algorithm 1. Then, $t=\theta_{B,i}/2$ is adopted to obtain the optimal value of the beamwidth parameter $\theta_{B,i}^{opt}$ of the system through conversion. The data transmission between the UAV and the ground users can make the system’s performance optimal.
**Algorithm 1:** The Proposed Fixed-Point Iteration (FPI) Algorithm**Initialization**$:\;\mathrm{Set}\;\mathrm{initial}\;\mathrm{value}\;t_{0}$, Convergence decision threshold ε, iterations k=1 **Repeat**:                 Calculate $t_{k}=g\left(t_{k-1}\right)$, according to the Formula (14) **Set**: k=k+1 **Until**$:\;\left|t_{k}-t_{k-1}\right|<\varepsilon$**Return**: $t^{\mathrm{opt}}=t_{k}$

## 5. Numerical Result

In terms of the proposed UAV-assisted clustering and beamwidth optimization method of wireless communication in this paper, the location information of ground users generated from the randn() function randomly was adopted and MATLAB was utilized to conduct the simulation experiment. The feasibility of the proposed algorithm for associating UAV with cluster users and optimizing directional antenna beamwidth is verified. In addition, the impact for various parameters on the downlink system performance of UAV-assisted wireless communication is discussed. The specific values of the parameters in the simulation are presented in Table 1.

**Figure 2:**
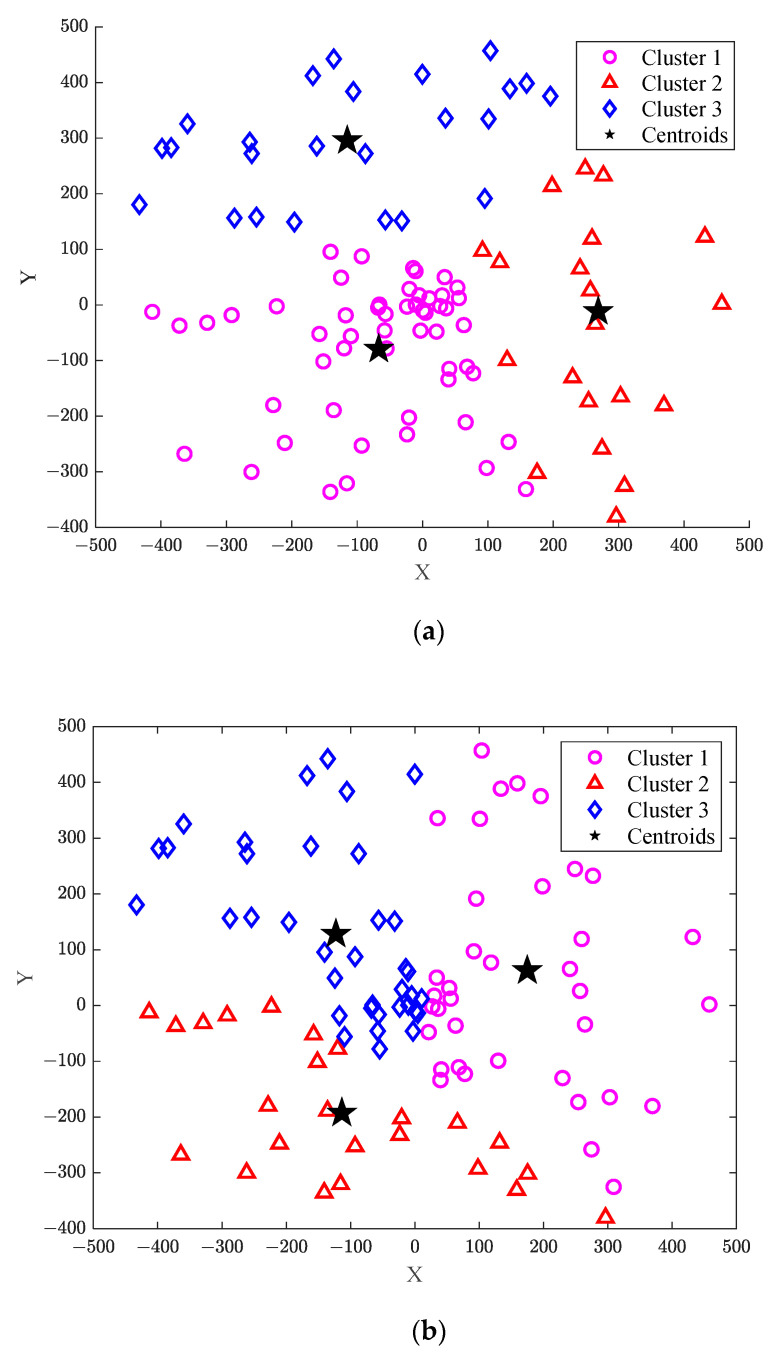
Comparison of clustering methods. (**a**) Traditional k-means clustering method; (**b**) improved k-means clustering method.

Figure 2 shows the comparison of two clustering methods when the cluster number is 3, where Figure 2a is obtained using the traditional k-means clustering method, while Figure 2b shows the results of the proposed improved k-means method. Table 2 presents the comparison of the number of users in the clusters with different clustering methods. As can be seen from Figure 2a, when the ground users are randomly distributed, the number of such users is 100 for the traditional k-means method, which can be divided into three clusters. The number of users in each cluster is, respectively, 22, 24, and 54. The number of users in cluster 3 is almost three times more than that in cluster 1. This indicates that the number of users in different clusters has great difference, which will impose a certain impact on the research of the subsequent energy efficiency. On the contrary, it can be seen from Figure 2b that the number of users in each cluster is, respectively, 39, 27, and 34, meaning that the number of users in each cluster is within the given limited range. Furthermore, the difference in the number of users in each cluster is small, making the clusters relatively uniform.

**Figure 3:**
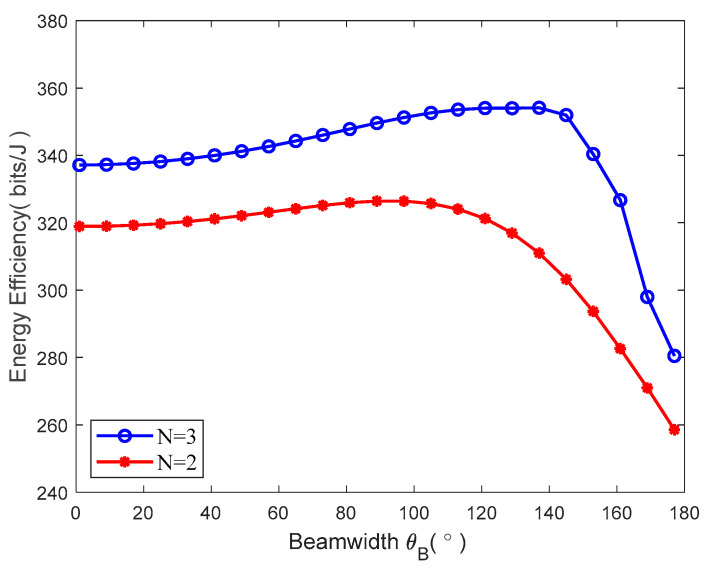
Beamwidth vs. energy efficiency.

**Figure 4:**
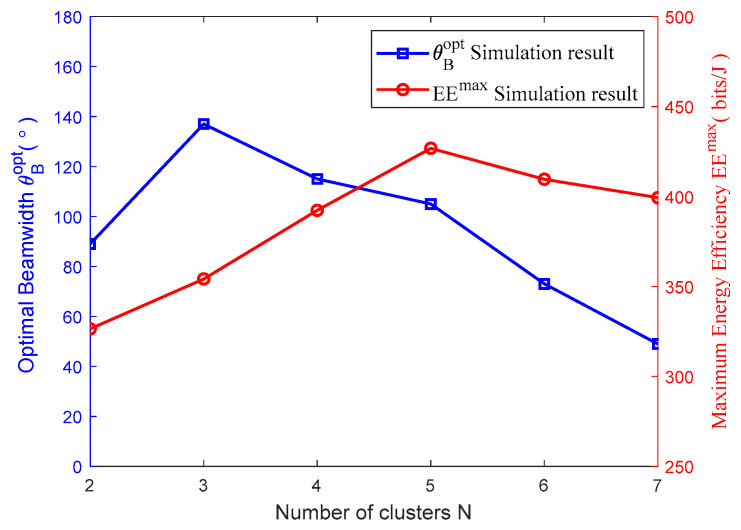
Optimal beamwidth, maximum energy efficiency vs. number of clusters.

Figure 3 displays the MATLAB simulation diagram between the beamwidth $\theta_{B}$ and system energy efficiency. When the beamwidth $\theta_{B}$ changes within (0,π), the energy efficiency firstly increases along with the increase in beamwidth, but when it keeps increasing subsequently, the energy efficiency decreases instead. This indicates that the optimal beamwidth can indeed maximize the energy efficiency, verifying the correctness of the previous analysis. Based on this, the relation schema of the beamwidth $\theta_{B}$ and the system’s energy efficiency under different clusters are compared. The red curve indicates that the ground users are divided into N=2 clusters, and the black one indicates that the users are divided into N=3 clusters. It can be seen that when the beamwidth is identical, the obtained system energy efficiency is slightly higher when the number of clusters is greater. But it can be seen from Figure 4 that such energy efficiency will decrease along with the monotonic increase in the number of clusters.

Figure 4 shows the relation schema between the optimal beamwidth $\theta_{B}^{opt}$, the corresponding maximum value $EE^{\max}$ of energy efficiency and the number of clusters N. As can be discovered from Figure 4, the obtained optimal beamwidth $\theta_{B}^{opt}$ through the maximization of system energy efficiency is different when the number of clusters is different. This demonstrates that the number of clusters indeed impacts the beamwidth. In addition, the maximum value $EE^{\max}$ of energy efficiency also depends on the number of clusters N and the optimal beamwidth $\theta_{B}^{opt}$. This also indicates that the system energy efficiency will not continue to increase along with the increase in the number of clusters. The increase in the number of clusters means the increase in the required number of UAVs. Consequently, the circuit power $P_{c}$ consumed by UAVs in the given area will increase as well. Thus, the increase in the total power consumed by UAVs will further compromise the system’s energy efficiency.

**Figure 5:**
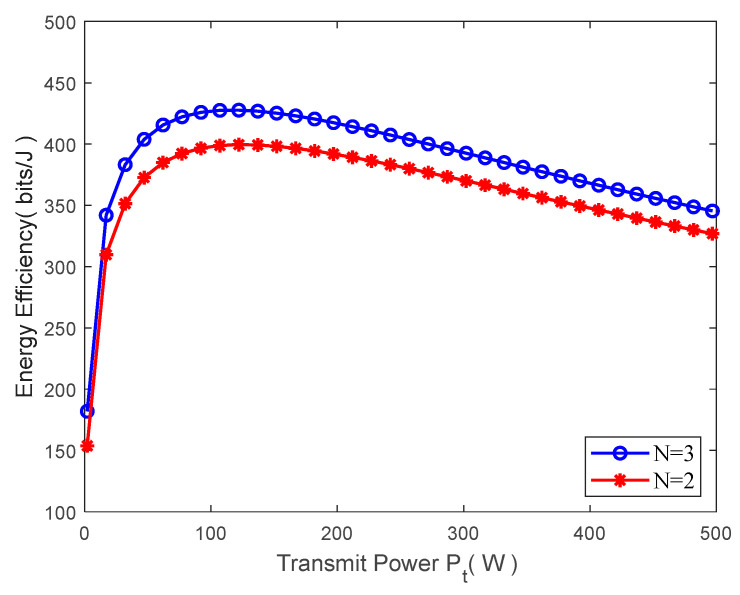
Transmit power vs. energy efficiency.

Figure 5 shows the relation between the total power $P_{t}$ of all users transmitted by UAVs and the system’s energy efficiency based on the premise that the beamwidth is fixed. When the transmitted power $P_{t}$ is lower, the energy efficiency will increase along with its increase; however, when it reaches a certain value, the total power consumed by the UAVs will increase too, thus leading to a decrease in energy efficiency. As can be seen from the simulation in Figure 5, except for the optimal beamwidth, the optimal transmitted power can also maximize the system’s energy efficiency.

**Figure 6:**
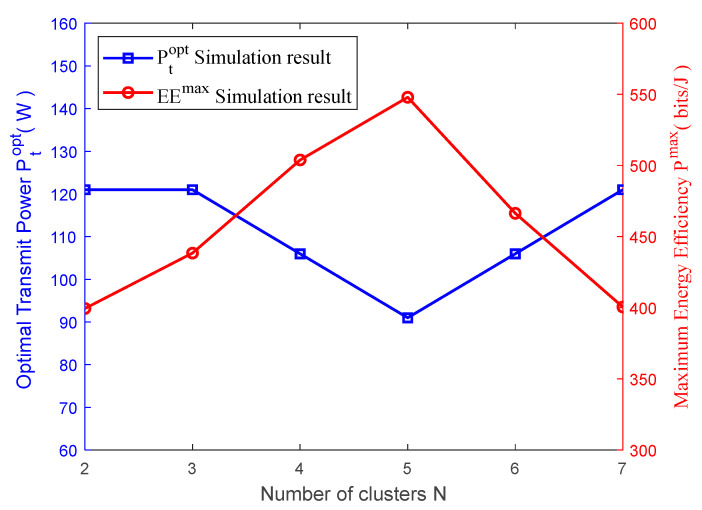
Optimal transmit power and maximum energy efficiency vs. number of clusters.

Figure 6 illustrates the change in optimal transmit power $P_{t}$ and the maximum energy efficiency $EE^{\max}$. As can be seen from Figure 6, with the constant increase in the number of clusters, the system energy efficiency shows a tendency for increasing at first and decreasing later due to the increase in the number of UAVs caused by the increase in the number of clusters. As a result, the total power consumed by the system increases, which compromises the energy efficiency.

According to the series of simulation results, except for the optimal beamwidth $\theta_{B}^{opt}$ and the number of clusters N, the maximum value of energy efficiency $EE^{\max}$ also has a certain relationship with the transmit power $P_{t}$ of UAV.

**Figure 7:**
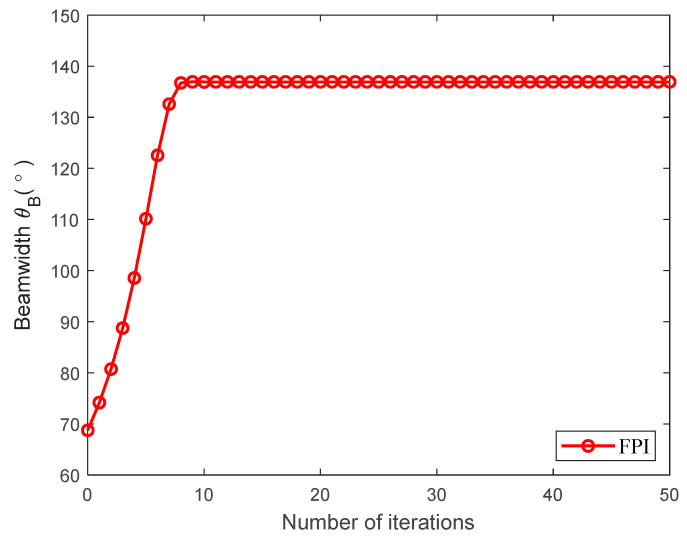
Convergence behavior of the proposed FPI algorithm.

Figure 7 verifies the convergence performance of the proposed FPI algorithm in this paper. From the simulation, it can be observed that the beamwidth of the directional antenna stabilizes after approximately nine iterations, indicating the convergence of the FPI algorithm proposed in this paper. Moreover, the proposed FPI algorithm also exhibits low computational complexity.

**Figure 8:**
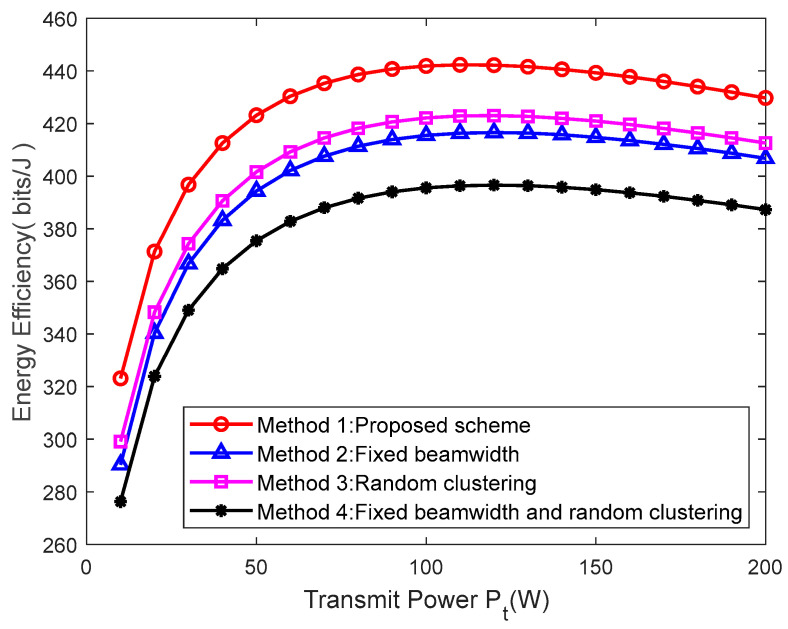
The different methods versus transmit power.

Finally, the performance of the proposed method was evaluated. In [21], the beamwidth of the UAV directional antenna is fixed, so Figure 8 compares the differences in UAV energy efficiency between the proposed method and the fixed beamwidth and random clustering scenarios. Among them, Method 1 involves the improved k-means algorithm and the FPI algorithm proposed in this article, Method 2 uses the fixed beamwidth and improved k-means algorithm, Method 3 uses the random clustering method and the FPI algorithm, and Method 4 uses the fixed beamwidth and random clustering method. From Figure 8, it can be seen that the energy efficiency of the proposed method is the highest. In addition, Method 2, using the improved k-means algorithm, and Method 3, using the FPI algorithm, both have higher energy efficiency than Method 4, indicating that the improved k-means algorithm and the FPI algorithm proposed in this paper can effectively improve the energy efficiency of UAVs.

## 6. Conclusions

The UAV-assisted clustering and beamwidth optimization method of wireless communication proposed in this paper achieves efficient data transmission between UAV and the ground users and raises the efficiency of the entire wireless communication system. According to the method proposed in this chapter, each user can only link with one UAV. Furthermore, the number of users in each cluster is limited. Based on the principle of “energy efficiency maximization”, the FPI algorithm is adopted to obtain the optimal beamwidth of the UAV-assisted wireless communication system. The simulations indicate that the method proposed in this paper can effectively raise the energy efficiency of the wireless communication system. In addition, the impact of the parameters, such as the beamwidth, number of clusters, and the transmitted power, on the energy efficiency is further discussed.

## Figures and Tables

**Table 1:** Simulation parameters.

Parameter	Value
Number of ground users M	100
Number of UAVs N	3
Area of region	500 × 500 (m^2^)
Carrier frequency $f_{c}$	2.4 GHz
Path loss index γ	2
LoS probability parameter α	9.61
NLoS probability parameter β	0.61
LoS Additional path loss $\eta_{\mathrm{LoS}}$	1
NLoS Additional path loss $\eta_{\mathrm{NLoS}}$	20
Noise power spectral density $N_{0}$	5 × 10^−15^ W/Hz
Bandwidth B	100 KHz
Maximum cluster size $M_{\max}$	40
Minimum cluster size $M_{\min}$	20

**Table 2:** Comparison of the number of users in the cluster by different clustering methods.

	Number of Users	Traditional K-Means Clustering Method	Improved K-Means Clustering Method
Cluster			
1		22	39
2		24	27
3		54	34

## Data Availability

Data are contained within the article.
